# Elevated Expression of miR-19b Enhances CD8^+^ T Cell Function by Targeting PTEN in HIV Infected Long Term Non-progressors With Sustained Viral Suppression

**DOI:** 10.3389/fimmu.2018.03140

**Published:** 2019-01-11

**Authors:** Lin-Bo Yin, Cheng-Bo Song, Jie-Fu Zheng, Ya-Jing Fu, Shi Qian, Yong-Jun Jiang, Jun-Jie Xu, Hai-Bo Ding, Hong Shang, Zi-Ning Zhang

**Affiliations:** ^1^NHC Key Laboratory of AIDS Immunology (China Medical University), Department of Laboratory Medicine, The First Affiliated Hospital of China Medical University, Shenyang, China; ^2^Key Laboratory of AIDS Immunology of Liaoning Province, The First Affiliated Hospital of China Medical University, Shenyang, China; ^3^Key Laboratory of AIDS Immunology, Chinese Academy of Medical Sciences, Shenyang, China; ^4^Collaborative Innovation Center for Diagnosis and Treatment of Infectious Diseases, Hangzhou, China

**Keywords:** CD8^+^T cells, long-term non-progressors, microRNA-19b, phosphatase and tensin homolog, HIV

## Abstract

Human immunodeficiency virus (HIV)-infected long-term non-progressors (LTNPs) are of particular importance because of their unique disease progression characteristics. Defined by the maintenance of normal CD4^+^T cells after more than 8 years of infection, these LTNPs are heterogeneous. Some LTNPs exhibit ongoing viral production, while others do not and are able to control viral production. The underlying basis for this heterogeneity has not been clearly elucidated. In this study, the miRNA expression profiles of LTNPs were assessed. The levels of microRNA-19b (miR-19b) were found to be significantly increased in peripheral blood mononuclear cells of LTNPs with lower rather than higher viral load. We made clear that miR-19b may regulate CD8^+^T cell functions in HIV infection, which has not been addressed before. Overexpression of miR-19b promoted CD8^+^T cell proliferation, as well as interferon-γ and granzyme B expression, while inhibiting CD8^+^T cells apoptosis induced by anti-CD3/CD28 stimulation. The target of miR-19b was found to be the “phosphatase and tensin homolog”, which regulates CD8^+^T cells function during HIV infections. Furthermore, we found that miR-19b can directly inhibit viral production in *in-vitro* HIV infected T cells. These results highlight the importance of miR-19b to control viral levels, which facilitate an understanding of human immunodeficiency virus pathogenesis and provide potential targets for improved immune intervention.

## Introduction

Human immunodeficiency virus (HIV) infected patients with atypical disease progression are of particular importance because they can provide important information regarding HIV pathogenesis and therapy. The first evidence of long-term non-progressors (LTNPs) was reported in 1993, showing that 15% of individuals infected with HIV maintain a CD4 count >500 cells/μl (1, 2). In 2005, study showed that ~1/300 HIV-infected patients had undetectable plasma HIV RNA loads without antiretroviral therapy (ART) (3). Described as “Elite Controllers” (ECs) in 2007, these patients maintained HIV RNA levels below 50 copies/ml for at least 1 year in the absence of ART (4–8). Although there is overlap between LTNPs and ECs, they are not identical. Most ECs exhibit minimal reductions in CD4^+^ T cells over time, although reductions are observed in some ECs. Furthermore, some LTNPs have ongoing viral production, while others do not (3, 5, 7, 9–11). Transcriptomic analysis showed that ECs with higher CD4^+^T cell numbers were indistinguishable from HIV-1-negative individuals. In contrast, ECs with lower CD4^+^ T cell numbers were similar to ART-treated patients, but different from HIV-1-negative individuals (12). Alterations in T cell homeostasis predict the loss of immunological control in ECs (13)4. However, viral control differs among LTNPs and the reason for this difference is currently unknown. LTNPs maintaining high viral loads are prone to long-term disease progression, with reduced life expectancy compared to HIV negative individuals (14, 15). Analysis of the underlying reason for the differing levels of viral control in LTNPs may facilitate an understanding of HIV pathogenesis and may provide for new approaches to immune intervention.

The level of virus control is affected by immunologic and virologic factors. MicroRNAs (miRNAs) may be a potential mechanistic factor involved in this process. It has been shown that miRNAs play important roles in the regulation of immune responses and viral production. In HIV infection, miRNAs can directly modulate viral production by targeting the HIV-1 genome or by indirectly modulating cellular genes that influence viral propagation (16–20). Dramatic advances have been made in understanding how miRNAs regulate the development and function of immune cells (21, 22), including CD8^+^T cells which are key players in the antiviral immune response (23, 24). It is established that miRNAs modulate the expression of numerous regulatory proteins required for the development, differentiation, and function of CD8^+^T cells (25). Studies have shown that, in HIV infection, miRNAs modulate the expression of key markers associated with T cell exhaustion or dysfunction, such as interleukin 10 and B lymphocyte-induced maturation protein-1 (26). We postulated that differential miRNA profiles may contribute to the divergent control of viral load in LTNPs by affecting viral production and/or CD8^+^T cell function. Although previous studies have identified miRNA profiles in HIV-infected ECs and LTNPs (16, 27–30), the role of miRNAs in the differential control of viral load in LTNPs has not been addressed.

In this study, miRNA expression profiles of LTNPs were assessed. The expression of miR-19b was found to be significantly increased in the peripheral blood mononuclear cells (PBMCs) of LTNPs with low (LTNP-Ls) compared to high levels (LTNP-Hs) of virus production. In addition, miR-19b promoted proliferation, and expression of interferon gamma (IFN-γ) and granzyme B, while inhibiting CD8^+^T apoptosis induced by anti-CD3/CD28 stimulation. The phosphatase and tensin homolog (PTEN) is a target of miR-19b regulating CD8^+^T cell function in HIV infection. Furthermore, we found that miR-19b directly inhibits viral production in HIV-infected T cells *in vitro*. Our results revealed a previously unknown mechanism of sustained viral control by miR-19b in a subtype of LTNPs, suggesting that miR-19b may be a novel target for immune intervention in HIV infection.

## Materials and Methods

### Study Population

In total, samples obtained from 27 LTNPs, six typical progressors (TPs), and four healthy controls (HCs) were analyzed. The LTNPs were HIV positive patients who maintained normal CD4^+^ T cell counts (CD4 >500 cells/μl) for >10 years (mean ± SD: 14.72 ± 1.79 years at the time of sample collection) without receiving ART (Supplemental Table 1). The TPs were ART naive HIV positive patients who progressed to CD4 counts < 500 cells/μl at 2.53 ± 0.95 years (Supplemental Table 2). The initial 347-miRNA array was performed in a training cohort, including nine LTNPs (age, mean ± SD: 41 ± 6 years; gender, eight males and one female), six TPs (age, mean ± SD: 30 ± 15 years; gender, six males) and four HCs (age, mean ± SD: 37 ± 6 years; gender, four males). From the training cohort, miRNAs differentially expressed in LTNPs with differing viral loads were detected in a subsequent validation group that included 18 LTNPs. Ethical approval was obtained from the First Hospital of China Medical University, Shenyang, China and written informed consent was provided by all participants.

### miRNA Array Analysis

Quantitative real-time polymerase chain reaction (qRT-PCR)-based high-throughput miRNA profiling was performed at QuantoBio Biotechnology Co. Ltd. (Beijing, China). Briefly, total RNA extracted from peripheral blood mononuclear cells (PBMCs) was isolated using TRIzol® (Invitrogen, Carlsbad, USA). *Escherichia coli* poly (A) polymerase was used to add adenines to the 3′ end of RNA molecules lacking a poly (A) tail. After oligo dT annealing, a universal tag was attached to the 3′ end of cDNAs during cDNA synthesis using retrotranscriptase Superscript III (Invitrogen). With this universal tag, a SYBR®-based qRT-PCR was performed using miRNA-specific forward primers and a reverse universal primer mix. Of note, U1 and U6 were used in the training cohort for normalization. The variation of change in the threshold cycle (CT, target-CT, and control) was evaluated and used as a relative qualitative value.

### RT-PCR Quantification of miRNA and mRNA

We extracted miRNAs from cells using the miRNeasy Micro kit (Qiagen, Hilden, Germany). The RNA was reverse transcribed using a Primpscript® RT reagent kit (TAKARA, Dalian, China) according to the instructions provided by the manufacturer. Subsequently, RT-PCR was performed using a SYBR® Premix Ex Taq™ II (TAKARA). The levels of miRNA were normalized to the U6 small nucleolar RNA and quantified through the relative quantification method (2^−ΔΔ*Ct*^), as previously described (31). Cellular total mRNA was isolated using the RNeasy Micro kit (Qiagen). The cDNA was generated using the Primpscript® RT reagent kit (TAKARA). The levels of mRNA were quantified through the SYBR® Premix Ex Taq™ II (TAKARA), normalized to GAPDH transcripts, and expressed using the relative quantification method (2^−ΔΔ*Ct*^). All primer sequences for the quantification of miRNA and mRNA are listed in Supplemental Table 3.

### Isolation of Cells

PBMCs were obtained by Ficoll–Hypaque density gradient centrifugation. If indicated, CD8^+^ or CD4^+^ T cells were further purified from isolated PBMCs by negative selection with magnetic beads using a CD8^+^ or CD4^+^ T cell Enrichment Kit (Cell purity >95%, Stem Cell Technologies, Vancouver, Canada). The following antibodies were used for immunostaining to isolate cell subtypes: FITC-CD3, APC-cy7-CD8, APC-CD4, PE-cy7-CD14 and 7-AAD (Biolegend, San Diego, CA, USA). CD4^+^ T cells (CD3^+^CD4^+^), CD8^+^ T cells (CD3^+^CD8^+^), and monocytes (CD3^−^CD14^+^) were selected from 7-AAD-negative live PBMCs using a FACSAria™ flow cytometer (BD Biosciences, Franklin Lake, NJ, USA).

### Cell Culture

The Jurkat human leukemia T cells, Clone-X cells, and primary cells were maintained in RPMI1640 media (HyClone, Logan, UT, USA) supplemented with 10% fetal bovine serum and 1% penicillin-streptomycin. The 293T cells were maintained in DMEM media (HyClone) supplemented with 10% fetal bovine serum and 1% penicillin-streptomycin.

### Transfection

Transfection of miRNAs to cell lines was achieved using Lipofectamine® 2000 (Invitrogen). Briefly, 20 μM miR-19b mimics or controls (GenePharma, Shanghai, China) were transfected to 293T cells, Jurkat cells, or Clone-X cells according to the protocol provided by the manufacturer. In primary cells, Lipofectamine® RNAiMAX (Invitrogen) was used for the transfection according to the protocol provided by the manufacturer. Briefly, 20 μM miR-19b mimics, inhibitors (GenePharma), or controls were transfected to isolated CD8^+^T or CD4^+^T cell-depleted PBMCs. In addition, isolated primary CD4^+^ T cells from healthy controls were transfected with 20 μM miR-19b mimics or controls. The forced reduction of phosphatase and tensin homolog (PTEN) was achieved by introducing 20 μM PTEN siRNA (Invitrogen) to isolated CD8^+^T cells. The siRNA control used in this experiment was non-specific Stealth RNAi® Negative Control Duplexes. The sequences of the mimics and inhibitors are listed in Supplemental Table 3.

### Proliferation Assays

After transfection (24 h), Jurkat cells and primary CD8^+^T cells were labeled with Cell Trace™ Violet (Thermo Fisher Scientific, Waltham, USA) for 15 min at 37°C according to the instructions provided by the manufacturer, washed with complete medium, and cultured (1 × 10^6^ cells/ml). Primary CD8^+^T cells were cultured in the presence of anti-CD3/CD28 (3 μg/ml; Gibco, New York, NY, USA). After incubation for 5 days, the dividing cells were analyzed using a BD LSR II flow cytometer and the FlowJo software.

### Detection of Apoptosis

After transfection (72 h), Jurkat cells were stained with PE-conjugated anti-Annexin V and 7-AAD (Biolegend). After transfection (24 h), primary CD8^+^T cells were stimulated using anti-CD3/CD28 (3 μg/ml). After stimulation (48 h), CD8^+^T cells were stained with PE-cy7-conjugated anti-CD3, APC-cy7-conjugated anti-CD8, PE-conjugated anti-Annexin V, and 7-AAD (Biolegend). The cells were analyzed using a BD LSR II flow cytometer and the FlowJo software.

### Cell Cycle Assay

Cell cycle phases were determined using the BD Cycletest™ Plus DNA Reagent Kit (BD Biosciences) according to the instructions provided by the manufacturer. In brief, Jurkat cells were cultured for 72 h after transfection. After transfection (24 h), primary CD8^+^T cells were cultured for 48 h under stimulation using anti-CD3/CD28 (3 μg/ml). The distribution of DNA content was determined using a BD LSR II flow cytometer and analyzed using the FlowJo software.

### Intracellular Staining of IFN-γ and Granzyme B

After transfection (24 h), primary CD8^+^T cells were stimulated using anti-CD3/CD28 (3 μg/ml) for 24 h. The protein transport inhibitor (GolgiStop; 1 μl/ml, BD Biosciences) was added to the culture for the last 6 h. The cells were stained with PE-cy7-conjugated anti-CD3 and APC-cy7-conjugated anti-CD8 (Biolegend). Subsequently, intracellular staining was performed by incubating the cells in 1X Perm/Wash Buffer for 15 min in the dark, followed by incubation with APC-conjugated anti-IFN-γ and FITC-conjugated anti-granzyme B for 30 min at 4°C. After staining, the cells were fixed in 1% formaldehyde. The intracellular expression of IFN-γ and granzyme B was determined using a BD LSR II flow cytometer and data were analyzed using the FlowJo software.

### IFN-γ ELISpot Assay

CD4^+^ T cells were depleted from PBMCs using anti-CD4 MAb-coated magnetic beads (Biolegend) as described in the instructions provided by the manufacturer. The Human IFN-γ ELISpot Kit (Mabtech, Nacka, Sweden) was used to detect the secretion of IFN-γ according to the instruction manual. After transfection (24 h), 2 × 10^5^ CD4^+^ T cell-depleted PBMCs were added per well (in duplicates) in a volume of 200 μl. The HIV-1 gag peptide pools (10 μg/ml, Sigma) were added for 20 h. Anti-CD3/CD28 (3 μg/ml) were used as a positive control, and negative controls consisted of cells without stimuli. The number of IFN-γ-secreting cells was calculated by subtracting the negative control (medium only) values. A positive response was defined as >50 spot-forming units/10^6^ PBMC.

### *In-vitro* Infection

Viral particles were produced by transfecting 293T cells with HIV-1 pNL4-3 plasmids and vesicular stomatitis virus glycoprotein (VSV-G) plasmids. Transfection of miR-19b mimics, pNL4-3 plasmids, and VSV-G plasmids into 293T cells was performed to detect the effects of miR-19 on HIV production. The levels of p24 in the supernatants were measured by ELISA (Biomedical Engineering Center of Hebei Medical University, Hebei, China) 2 days later. For the infection of Clone-X cells, the cells were transfected with miR-19b mimics for 24 h and subsequently infected with VSV-G pseudotyped HIV-1 (NL4-3) virus. GFP^+^ cells were detected by flow cytometry 48 h after infection.

Replication-competent HIV-1 isolate was used to test the effects of miR-19b in primary CD4^+^ T cells. Isolated primary CD4^+^ T cells from healthy controls were transfected with miR-19b mimics or controls. After transfection (24 h), the cells were stimulated using anti-CD3/CD28 (3 μg/ml). A cryopreserved primary HIV-1 isolate—obtained by a co-culture using mixed PBMCs from an HIV-1-infected patient and a healthy donor—was thawed and added to the cells. The supernatant was collected after 3 days of infection and the levels of p24 in the supernatants were measured by ELISA.

### Statistical Analysis

Principal component analysis (PCA) was used (Origin 9.1 software) to analyze the distribution of miRNAs in HIV-infected patients with differing disease progression. The non-parametric Mann–Whitney test was used to determine differences between LTNPs with a relatively high viral load (>1,000 copies/ml) (LTNP-Hs) and LTNPs with relative control of viral load (< 1,000 copies/ml) (LTNP-Ls). A paired *t*-test was used to analyze differences in CD8^+^T cell function between groups. Data analysis was performed using the SPSS 21.0 and GraphPad Prism Version 5.0 software packages. A *P* < 0.05 was considered statistically significant.

## Results

### miRNA Profiles Distinguish LTNPs With Different Virus Levels

**Figure 1:**
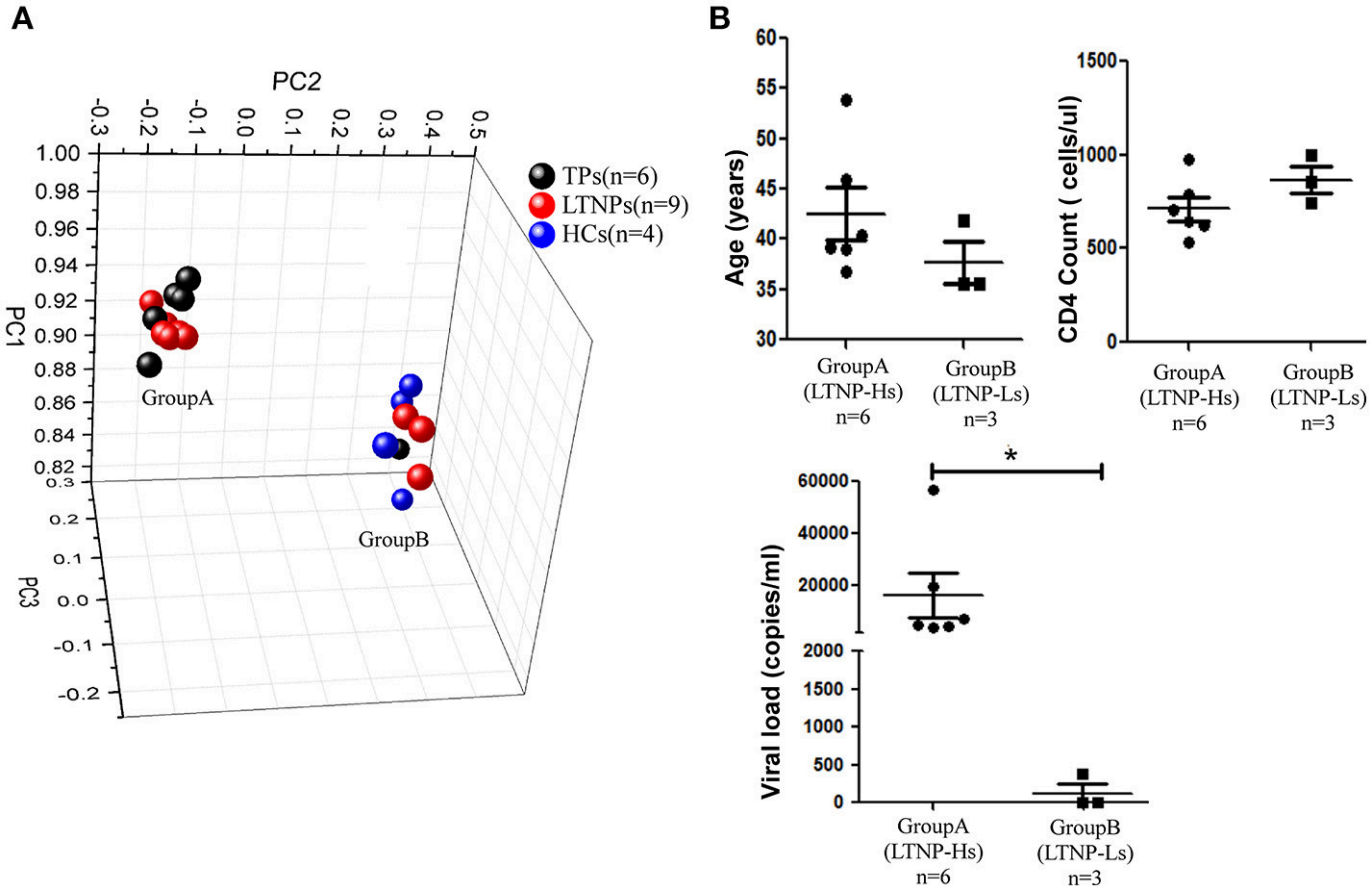
miRNA profiles distinguish LTNPs with different levels of virus. **(A)** Principal component analysis (PCA) plot of miRNA expression data from LTNPs, TPs, and HCs in the training cohort. Nine LTNPs were divided into two groups, one of which was very close to the TPs (Group A, *n* = 6) and another that was intertwined with the HCs (Group B, *n* = 3). **(B)** Comparison of age, CD4 counts, and viral load between Group A and Group B of LTNPs. ^*^P < 0.05.

A training cohort was formed including nine LTNPs, six TPs, and four HCs to identify the miRNA profiles of LTNPs. Using qRT-PCR-based arrays, the expression levels of 347 miRNAs were quantified. Based on an unsupervised PCA of all array data, the six TPs, nine LTNPs and four HCs were segregated into two groups (Figure 1A). All the HCs were clustered in one group. Most of the TPs were clustered in the other group, except one TP with a relatively low viral load (< 1,000 copies/ml), indicating that HIV infection alters miRNAs. This finding was consistent with those reported in previous studies (30, 32, 33). Interestingly, the nine LTNPs were divided into two groups, one of which was very close to the TPs (Group A, *n* = 6) and another that was intertwined with the HCs (Group B, *n* = 3) (Figure 1A). We subsequently sought to identify differences between the two groups of LTNPs. By comparison of clinical characteristics (i.e., age, number of CD4^+^T cells, and viral loads), we found that viral load was the only significantly different parameter between the two groups of LTNPs (*P* = 0.024, Figure 1B). Six LTNPs in Group A, whose miRNA profiles were similar to those of TPs, had a relatively high level of viral load (>1,000 copies/ml, hereinafter referred to as “LTNP-Hs”). Three LTNPs in Group B, whose miRNA profiles were similar to those of HCs, had relative control of viral load (VL < 1,000 copies/ml, hereinafter referred to as “LTNP-Ls,” Supplemental Table 1). The results of the unsupervised PCA suggest that the expression of miRNAs can distinguish LTNP-Hs and LTNP-Ls, and may account for the differing viral loads observed in LTNPs.

### Expression of miR-19b Is High in LTNP-Ls in Comparison With That in LTNP-Hs

**Figure 2:**
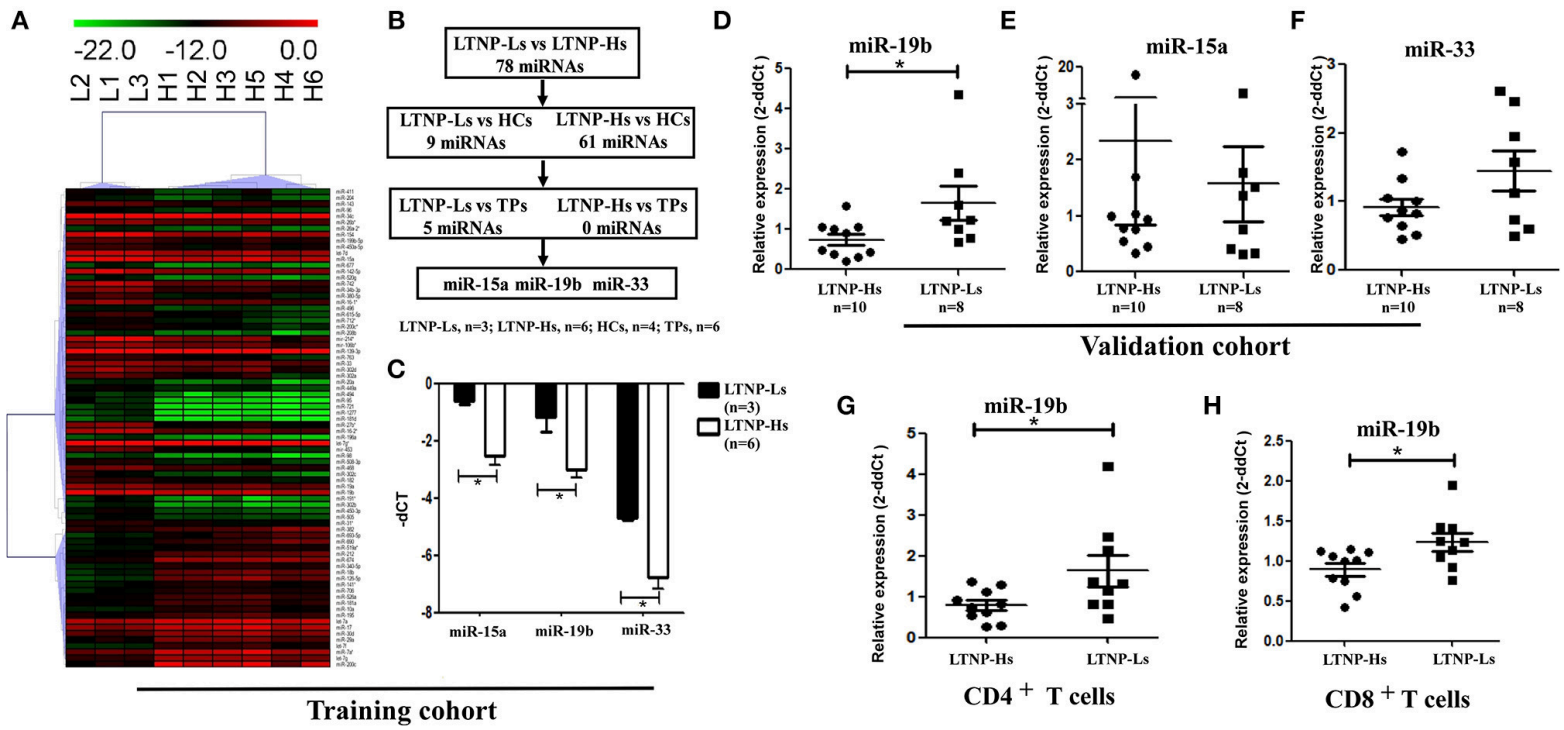
Expression of miR-19b is high in LTNP-Ls compared with that observed in LTNP-Hs. **(A)** Heatmap demonstrating 78 miRNAs differentially expressed between LTNP-Hs (*n* = 6) and LTNP-Ls (*n* = 3) in the training cohort (Benjamini–Hochberg false discovery rate-adjusted *P* < 0.05 and fold change >2). Hierarchical clustering of change in the threshold cycle (ΔCT) was performed using the complete linkage method and Pearson correlation coefficient. **(B)** The protocol for the selection of candidate miRNA from the training cohort. Among the 78 miRNAs differentially expressed between LTNP-Hs (*n* = 6) and LTNP-Ls (*n* = 3), 70 miRNAs differentially expressed between LTNPs and HCs (*P* < 0.05) were excluded. Subsequently, five differentially expressed miRNAs between LTNPs and TPs were excluded. Three candidate miRNAs, namely miR-15a, miR-19b, and miR-33 were selected. **(C)** Comparison of the three candidate miRNAs between LTNP-Ls (*n* = 3) and LTNP-Hs (*n* = 6) in the training cohort. **(D,F)** Relative expression of miR-19b **(D)**, miR-15a **(E)** and miR-33 **(F)** in PBMCs obtained from LTNP-Ls (*n* = 8) and LTNP-Hs (*n* = 10) in the subsequent validation group. **(G,H)** CD4^+^ and CD8^+^T cells from LTNPs were sorted through flow cytometry. The expression of miR19b in CD4^+^ **(G)** and CD8^+^ T **(H)** cells was compared between LTNP-Ls (*n* = 9, one from training cohort, eight from validation cohort) and LTNP-Hs (*n* = 10, two from training cohort, eight from validation cohort) using qRT-PCR. ^*^P < 0.05.

Subsequently, the differential expression of miRNAs in LTNP-Hs and LTNP-Ls was assessed. The miRNAs were determined to be significantly differentially expressed in LTNP-Hs and LTNP-Ls with a Benjamini–Hochberg false discovery rate-adjusted *P* < 0.05. We found that 78 miRNAs were differentially expressed with >2-fold change between the three LTNP-Ls and six LTNP-Hs in the training cohort (adj. *P* < 0.05, Supplemental Table 4). Among those, 55 miRNAs were upregulated and 23 were downregulated in LTNP-Ls compared with that in LTNP-Hs. Using an unsupervised clustering method, 78 miRNAs accurately distinguished and clustered LTNP-Hs and LTNP-Ls (Figure 2A). A total of 75 miRNAs with differential expression levels between LTNP-Hs or LTNP-Ls and HCs or TPs (*P* < 0.05) were excluded to identify miRNAs that can uniquely differentiate LTNP-Hs from LTNP-Ls (Figure 2B; Supplemental Table 4). This exclusion was carried out because these miRNAs may reflect differences caused by HIV infection or the stages of infection. Only three miRNAs which were differentially expressed between LTNP-Hs and LTNP-Ls in the training cohort were selected, including miR-15a (*P* = 0.024), miR-19b (*P* = 0.048), and miR-33 (*P* = 0.024, Figure 2C).

The expression levels of these three miRNAs were assessed in a subsequent validation group, including ten LTNP-Hs and eight LTNP-Ls (Supplemental Table 1). In the validation cohort, only miR-19b was verified to be highly expressed in LTNP-Ls compared with LTNP-Hs (*P* = 0.034, Figures 2D–F). Subsequently, CD8^+^, CD4^+^ T cells, and monocytes from LTNPs were sorted to identify the cell subtypes in which the expression of miR-19b was altered. We found that the expression of miR-19b decreased in CD4^+^T cells and CD8^+^ T cells in LTNP-Hs (*n* = 10) compared with that observed in LTNP-Ls (*n* = 9) (*P* = 0.041, Figure 2G; *P* = 0.028, Figure 2H). There were no differences observed in the expression of miR-19b in monocytes between the two groups (data not shown).

### miR-19b Promotes Proliferation and Expression IFN-γ, and Inhibits the Apoptosis of Primary CD8^+^T Cells

**Figure 3:**
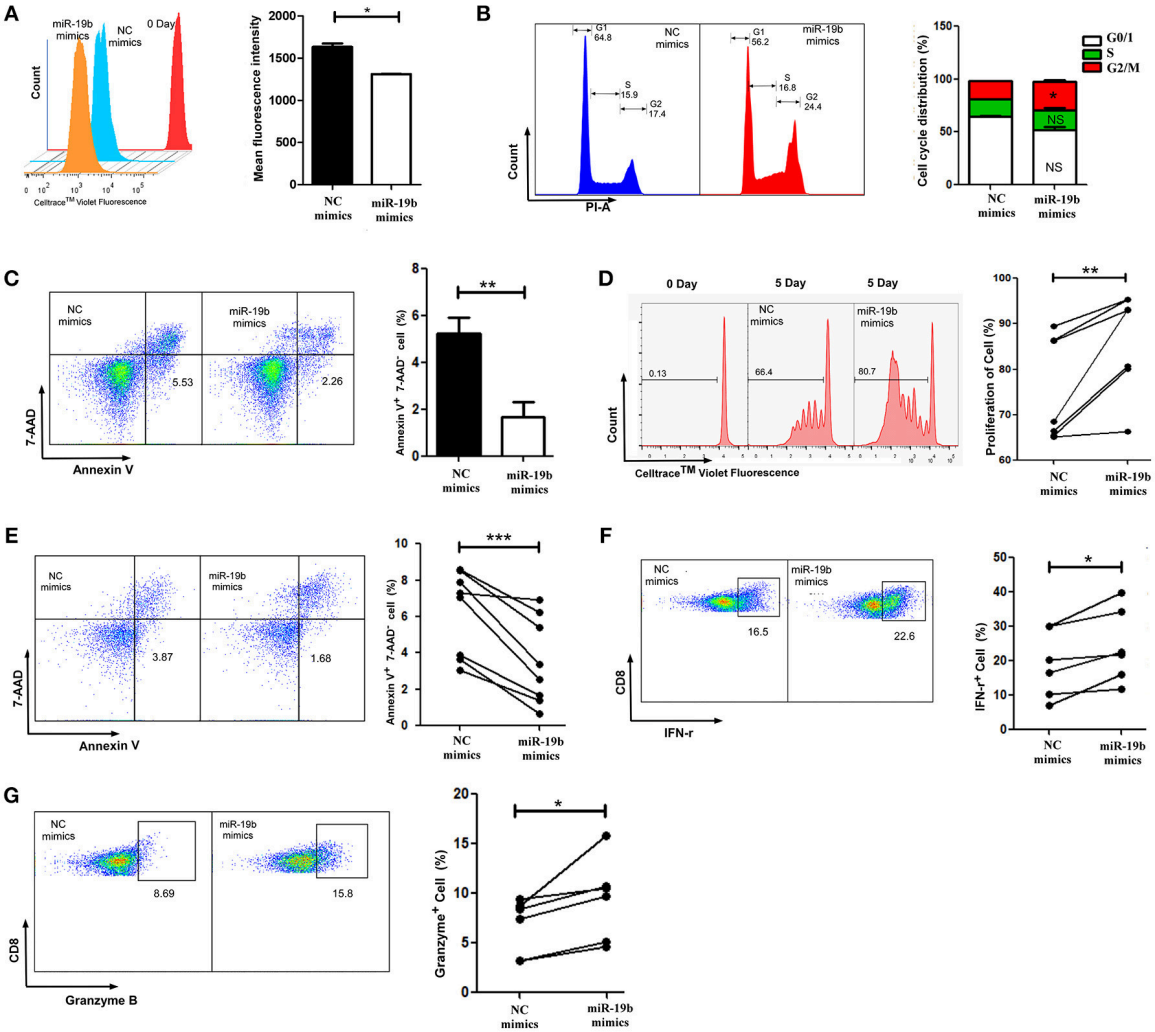
Overexpression of miR-19b regulates the function of CD8^+^ T cells from healthy controls. Jurkat cells were transfected with miR-19b mimics using Lipofectamine® 2000. **(A)** After transfection (24 h), Jurkat cells were labeled with Cell Trace™ Violet. After incubation for 5 days, the dividing cells were analyzed. **(B)** After transfection (72 h), the cell cycle of Jurkat cells was determined. **(C)** After transfection (72 h), Jurkat cells were stained with Annexin V and 7-AAD and the apoptosis of the cells was determined. **(D–G)** CD8^+^ T cells from healthy controls were sorted and transfected with miR-19b mimics or controls using Lipofectamine® RNAiMAX. **(D)** After transfection (24 h), CD8^+^T cells were labeled with Cell Trace™ Violet and stimulated with anti-CD3/CD28 (3 μg/ml) for 5 days, the proliferation of CD8^+^T cells was determined. **(E)** After transfection (24 h), CD8^+^T cells were stimulated using anti-CD3/CD28 (3 μg/ml) for 48 h and the rate of apoptosis were determined. **(F,G)** After transfection (24 h), CD8^+^ T cells were stimulated using anti-CD3/CD28 (3 μg/ml) for 24 h and expression of intracellular IFN-γ **(F)** and granzyme B **(G)** was determined. In each part, representative flow cytometry data and comparisons of the parameters between miR-19b-overexpressing cells and controls are shown. *n* = 7 for each group in **(D)**, *n* = 8 for each group in **(E)**, *n* = 6 for each group in **(F,G)**. ^*^P < 0.05. ^**^P < 0.01, ^***^P < 0.001, respectively.

As the most important immune effector cell population, CD8^+^T cells play a key role in anti-HIV immune responses. We hypothesized that miR-19b may contribute to the control of the virus in LTNP-Ls by regulating CD8^+^T cell function. This was the first study addressing this question. Initially, we overexpressed miR-19b in Jurkat cells by transfection with miRNA mimics to investigate the effect of miR-19b on lymphocyte proliferation and apoptosis. After transfection (48 h), miR-19b was highly expressed (Supplemental Figure 1). After 5 days, the proliferation of miR-19b-overexpressing cells was significantly increased compared with that observed in the control group, suggesting that miR-19b promotes cell proliferation (*P* = 0.013, Figure 3A). Overexpression of miR-19b significantly accelerated the cell cycle (*P* = 0.033, Figure 3B). Furthermore, the percentage of Annexin V^+^ 7-AAD^−^ apoptotic cells was lower following the overexpression of miR-19b (*P* = 0.005, Figure 3C). These data indicate that miR-19b promotes proliferation and inhibits apoptosis of T cell lines.

We subsequently assessed the role of miR-19b in regulating the function of primary CD8^+^T cells by transfection of miR-19b mimics into CD8^+^T cells obtained from HCs. We found that overexpression of miR-19b significantly promoted the proliferation of CD8^+^T cells after anti-CD3/CD28 stimulation (*P* = 0.010, Figure 3D). Similarly, forced expression of miR-19b significantly inhibited apoptosis of CD8^+^T cells after stimulation of the anti-CD3/CD28 (*P* = 0.009, Figure 3E). CD8^+^T cells were sorted using magnetic beads to study the effect of miR-19b on the cytotoxic function of CD8^+^T cells. Overexpression of miR-19b in CD8^+^T cells significantly increased the intracellular levels of IFN-γ and granzyme B after stimulation of the anti-CD3/CD28 (*P* = 0.006, Figure 3F; *P* = 0.002, Figure 3G, respectively). These results demonstrate that miR-19b promotes cell proliferation, and the expression of IFN-γ and granzyme B in CD8^+^T cells. Moreover, it inhibits the apoptosis of CD8^+^T cells after stimulation of the anti-CD3/CD28.

### miR-19b Regulates CD8^+^ T Cell Function via Expression of PTEN

**Figure 4:**
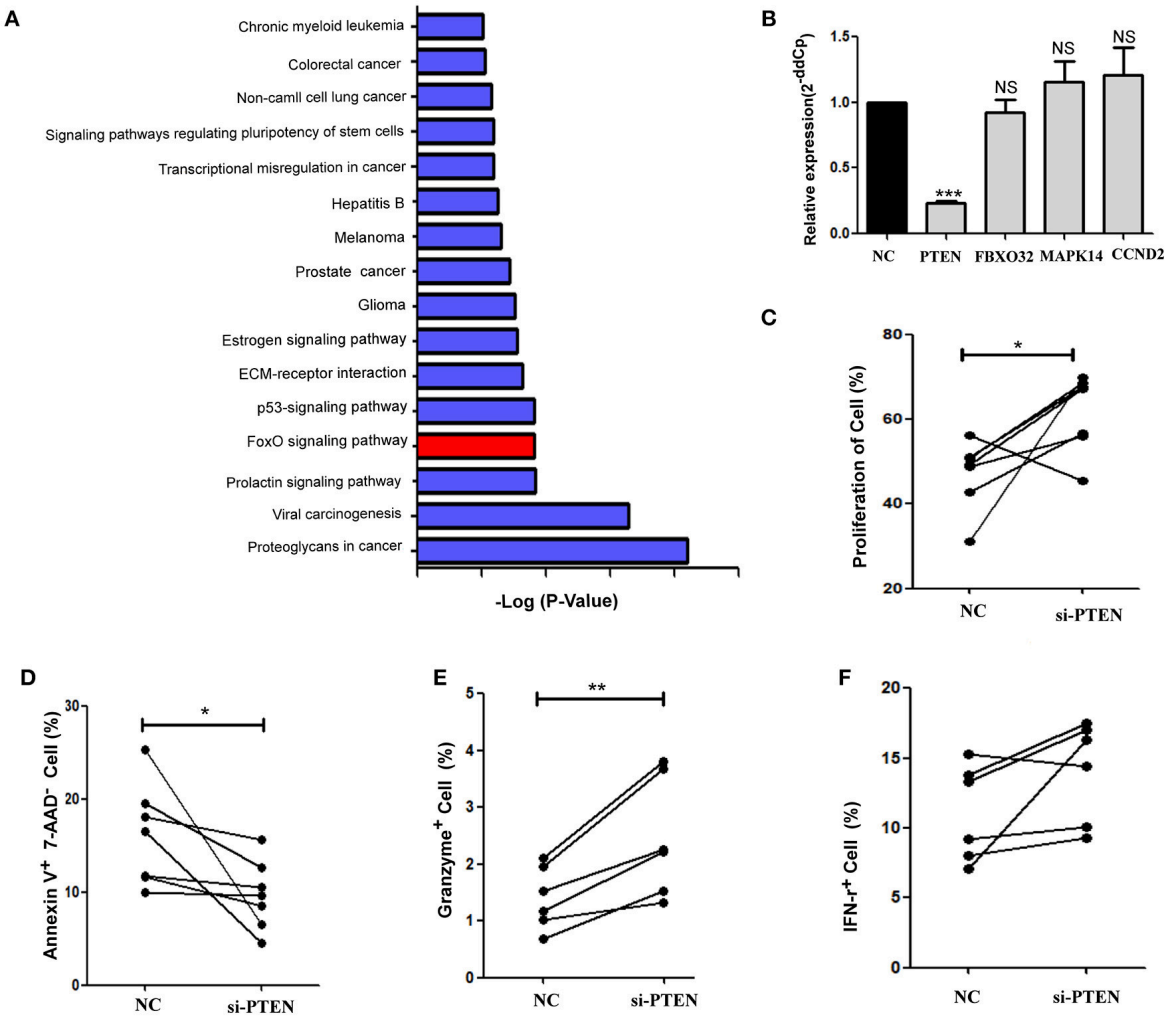
PTEN is a potential target of miR-19b. **(A)** Cell signaling pathways involving miR-19b target genes were assessed through a bioinformatics analysis (http://diana.imis.athena-innovation.gr/). The FOXO signaling pathway (Red) is a major cellular signaling pathway playing a key role in cell cycle regulation. **(B)** The expression of miR-19b was enhanced by mimics in Jurkat cells. Four genes in the FOXO pathway were detected through qRT-PCR. **(C–F)** The expression of PTEN was inhibited by introducing 20 μM PTEN siRNA to isolated CD8^+^T cells. **(C)** After transfection (24 h), CD8^+^T cells were labeled with Cell Trace™ Violet and stimulated using anti-CD3/CD28 (3 μg/ml) for 5 days. The proliferation of CD8^+^T cells was determined. **(D)** After transfection (24 h), CD8^+^ T cells were stimulated using anti-CD3/CD28 (3 μg/ml) for 48 h and the rate of apoptosis of CD8^+^ T cells was determined. **(E,F)** After transfection (24 h), CD8^+^ T cells were stimulated using anti-CD3/CD28 (3 μg/ml) for 24 h and the intracellular expression of IFN-γ **(E)** and granzyme B **(F)** was determined. *n* = 6 for each group in **(C)**, *n* = 7 for each group in **(D)**, *n* = 6 for each group in **(E,F)**. ^*^P < 0.05. ^**^P < 0.01. ^***^P < 0.001, respectively.

Cell signaling pathways involving miR-19b target genes were assessed through a bioinformatics analysis (http://diana.imis.athena-innovation.gr/) to further explore the molecular mechanistic basis of miR-19b regulation (Figure 4A). The FOXO signaling pathway plays critical roles in cell cycle regulation and is the main cell signaling pathway in which miR-19b target genes are involved. Through the detection of several key molecules in the FOXO pathway using qRT-PCR, we found that overexpression of miR-19b in Jurkat cells significantly reduced the expression level of PTEN (*P* < 0.001, Figure 4B). The direct regulation of PTEN by miR-19b was demonstrated using a Luciferase reporter assay, qRT-PCR, and western blotting (34–38). Considering that PTEN is closely associated with cell proliferation and cell cycle (39), we sought to determine the effect of miR19b on the function of CD8^+^T cells by regulating the expression of the target gene PTEN.

We reduced the expression of PTEN in primary HCs CD8^+^T cells using specific siRNAs (Supplemental Figure 1). Cell proliferation was promoted and apoptosis was inhibited via the knockdown of PTEN in comparison with the negative control group (*P* = 0.040 Figure 4C; *P* = 0.047, Figure 4D, respectively). Moreover, the secretion of granzyme B by CD8^+^ T cells was significantly increased (*P* = 0.006, Figure 4E) and that of IFN-γ showed an increasing trend (*P* = 0.094, Figure 4F) after stimulation of the anti-CD3/CD28, in response to the suppression of PTEN. These findings demonstrate that inhibition of PTEN, which is a potential target of miR-19b, exerts similar effects on CD8^+^T cell function to those observed following the overexpression of miR-19b.

### miR-19b Regulates the Function of CD8^+^ T Cells From HIV-Infected Patients

**Figure 5:**
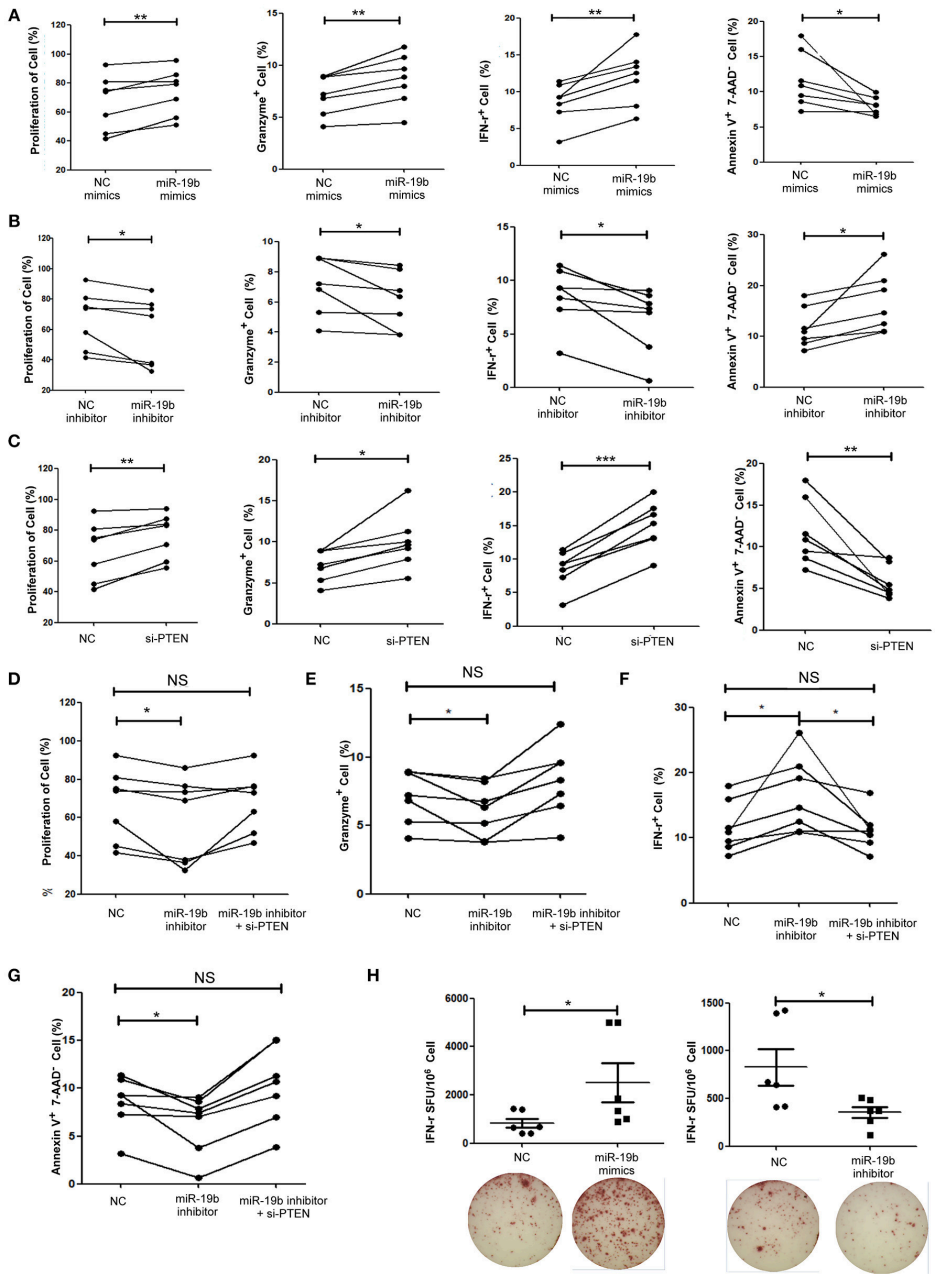
miR-19b regulates CD8^+^T cell function in HIV-infected patients. CD8^+^ T cells from HIV-infected treatment-naive patients were sorted. **(A)** CD8^+^ T cells were transfected with miR-19b mimics using Lipofectamine® RNAiMAX. After transfection (24 h), CD8^+^ T cells were stimulated using anti-CD3/CD28 (3 μg/ml). Proliferation (day 5), intracellular expression of IFN-γ and granzyme B (day 1), and apoptosis (day 2) were compared between miR-19b-overexpressing CD8^+^ T cells and controls. **(B)** CD8^+^ T cells were transfected with miR-19b inhibitors using Lipofectamine® RNAiMAX. After transfection (24 h), CD8^+^ T cells were stimulated using anti-CD3/CD28 (3 μg/ml). Proliferation (day 5), intracellular expression of IFN-γ and granzyme B (day 1), and apoptosis (day 2) were compared between miR-19b-overexpressing CD8^+^T cells and controls. **(C)** The expression of PTEN was inhibited by introducing 20 μM PTEN siRNA to isolated CD8^+^ T cells. Proliferation (day 5), intracellular expression of IFN-γ and granzyme B (day 1), and apoptosis (day 2) were compared between PTEN-inhibited CD8^+^ T cells and controls. **(D–G)** CD8^+^ T cells were transfected with a miR-19b inhibitor and PTEN siRNAs simultaneously. After transfection (24 h), CD8^+^ T cells were stimulated using anti-CD3/CD28 (3 μg/ml). Proliferation **(D)**, apoptosis **(G)**, and intracellular expression of granzyme B **(E)** and IFN-γ **(F)** were compared. **(H)** CD4^+^ T cells were depleted from PBMCs and transfected with miR-19b mimics or inhibitors. An IFN-γ ELISPOT assay was performed 24 h after transfection. Spot-forming units were compared between miR-19b-overexpressing/inhibited cells and controls. *n* = 7 for each group in **(A–G)**, *n* = 6 for each group in **(H)**. ^*^P < 0.05. ^**^P < 0.01. ^***^P < 0.001, respectively.

The function of miR-19b in CD8^+^T cells from 7 HIV-infected patients (Supplemental Table 5) was also studied. Primary CD8^+^T cells from HIV-infected patients were sorted. Following the overexpression of miR-19b (Supplemental Figure 1), CD8^+^T cells showed a significant increase in proliferation (*P* = 0.010), and secretion of IFN-γ (*P* = 0.010) and granzyme B (*P* = 0.003) after stimulation of the anti-CD3/CD28 (Figure 5A). Apoptosis of CD8^+^T cells was significantly reduced in comparison with the controls (*P* = 0.040, Figure 5A). Furthermore, the expression of miR-19b was inhibited by transfection of miR-19b inhibitors into CD8^+^ T cells from HIV patients (Supplemental Figure 1). Contrary to the miRNA overexpression results, the proliferation of CD8^+^ T cells was reduced (*P* = 0.043, Figure 5B), the secretions of IFN-γ (*P* = 0.023) and granzyme B (*P* = 0.049) were reduced, and the apoptosis of CD8^+^T cells was increased (*P* = 0.035, Figure 5B).

Subsequently, the expression of PTEN in primary CD8^+^T cells was inhibited using siRNA to verify that PTEN was a miR-19b target gene involved in the regulation of CD8^+^T cell function in HIV infection. Consistent with the findings reported in healthy patients, inhibition of PTEN resulted in an increase in proliferation (*P* = 0.005) and secretion of IFN-γ (*P* < 0.001) and granzyme B (*P* = 0.012), as well as a decrease in CD8^+^T cell apoptosis (*P* = 0.006) in HIV-infected patients (Figure 5C). Furthermore, siRNA was used to suppress the expression of PTEN in miR-19b-low-expressing CD8^+^ T cells to verify that miR-19b affects CD8^+^ T cell function through regulation of PTEN. Following the downregulation of PTEN, there was no statistical difference detected in proliferation, expression of IFN-γ and granzyme B, or apoptosis compared with controls. These data suggest that downregulation of PTEN antagonizes the effect of miR-19b inhibitors on the function of CD8^+^T cells in HIV patients (Figures 5D–G).

An IFN-γ ELISPOT assay was performed to further confirm the effect of miR-19b on HIV specific CD8^+^T cells. We found that overexpression of miR-19b significantly increased the secretion of IFN-γ by gag peptide-stimulated CD8^+^T cells (*P* = 0.041). Of note, the secretion of IFN-γ was significantly reduced in response to suppression of the expression of miR-19b by inhibitors (*P* = 0.020, Figure 5H). These data suggested that miR-19b augments the function of HIV specific CD8^+^T cells.

### miR-19b Inhibits Viral Replication in *in-vitro* HIV-Infected T Cells

**Figure 6:**
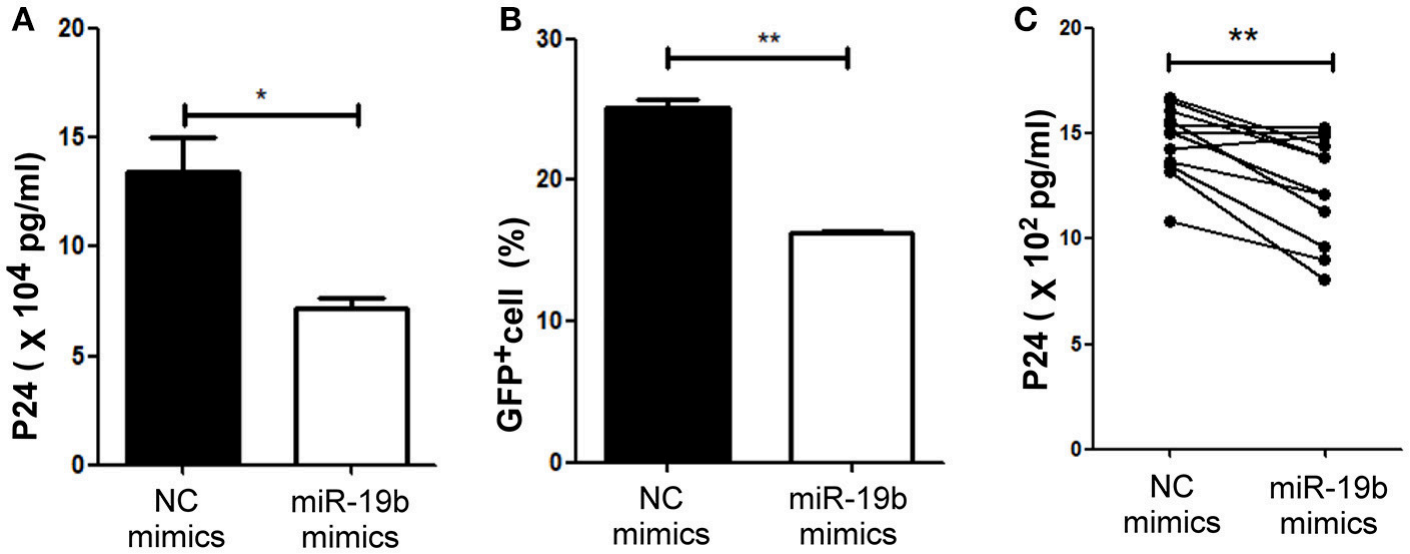
miR-19b inhibits viral replication in *in-vitro* HIV-infected T cells. **(A)** Mimics of miR-19b and the pseudoviral plasmid NL4-3 were co-transfected into 293T cells and the level of P24 in the supernatants was measured through ELISA. **(B)** Clone-X cells were transfected with miR-19b mimics for 24 h and subsequently infected with vesicular stomatitis virus glycoprotein (VSV-G) pseudotyped HIV-1 (NL4-3) virus. GFP^+^ cells were detected through flow cytometry 48 h after infection. **(C)** Primary CD4^+^ T cells isolated from healthy controls were transfected with miR-19b mimics or controls. After transfection (24 h), the cells were stimulated using anti-CD3/CD28 (3 μg/ml). A cryopreserved primary HIV-1 isolate was added to the cells and the levels of p24 in the supernatants were measured after 3 days of infection through ELISA. *n* = 12 for each group in **(C)** ^*^P < 0.05. ^**^P < 0.01.

Lastly, we hypothesized that miR-19b may play a role in the direct inhibition of HIV replication, besides its regulation of CD8^+^T cells. The levels of miR-19b in the plasma have been reported to be associated with CD4^+^ T cell counts, indicating that miR-19b may be a biomarker for the monitoring of the HIV immune status (40). However, its direct effects on HIV viral production have not been reported. We found that overexpression of miR-19b by mimics reduced the production of HIV. Following the co-transfection of miR-19b mimics and the pNL4-3 plasmid into 293T cells, the expression level of P24 in supernatants was lower than that in the control group after 48 h of culture (*P* = 0.040, Figure 6A). Furthermore, Clone-X cells were infected with the same titer of HIV pseudovirus, the percentage of HIV-positive cells overexpressing miR-19b was lower compared with that in the control group (*P* = 0.007, Figure 6B). Finally, we infected primary CD4^+^ T cells from normal controls using replication-competent HIV-1 virus isolates and found that overexpression of miR-19b inhibited the production of HIV (*P* = 0.001, Figure 6C). These data demonstrated that, besides its role in the regulation of CD8^+^T cells, miR-19b inhibits viral production, leading to lower viral levels.

## Discussion

LTNPs are defined by the maintenance of normal CD4^+^T cells counts for more than 8 years after infection. However, LTNPs exhibit heterogeneity in their viral loads. LTNPs maintaining high viral loads are prone to long-term progression, with reduced overall life expectancy vs. healthy individuals. However, the factors involved in the differential control of viral levels in LTNPs have not been identified. In this study, we found that miR-19b is highly expressed in LTNP-Ls vs. LTNP-Hs. It was shown that miR-19b influences the low viral load of LTNP-Ls by promoting the function of CD8^+^T cells in HIV infection and directly inhibiting viral production of HIV.

Factors contributing to the different levels of CD4^+^T cells in ECs have already been studied (12, 13). However, thus far, the underlying mechanistic basis for the differing levels of viral control in LTNPs has not been investigated. Considering the important role of miRNAs in regulating immune responses and viral replication, we performed a miRNA profiling analysis of HIV-infected individuals. The miRNA profiles distinguished LTNPs with differing viral loads. LTNPs whose miRNA profiles were similar to those of TPs exhibited a relatively high viral load. In contrast, LTNPs with miRNA profiles similar to those of HCs showed relatively well-controlled viral loads. Based on the results of the miRNA array analysis and subsequent validation, miR-19b was verified to be highly expressed in LTNP-Ls compared with LTNP-Hs. In HIV infection, plasma miR-19b was associated with CD4^+^ T cell counts and may be a useful biomarker for monitoring the HIV immune status (40). Our study showed that the expression of miR-19b is significantly different between LTNP-Hs and LTNP-Ls. We speculated that miR-19b contributes to the control of viral load in LTNP-Ls by affecting viral production and/or regulating immune cell function.

CD8^+^T cells play a crucial role in the control of HIV replication by direct cytolysis of infected cells and production of secreted factors (41, 42). We assessed the role of miR-19b in maintaining low levels of virus through the regulation of CD8^+^T cell function. Studies based on a lymphocytic choriomeningitis virus mouse model showed that knockout of the entire miR-17-92 cluster (miR-17, miR-18a, miR-19a, miR-20a, miR-19b, and miR-92a) impairs effector CD8^+^ T cell proliferation. In contrast, overexpression of the entire cluster promotes effector CD8^+^ T cell expansion and skews the differentiation of effector CD8^+^T cells to terminal effector cells (43, 44). The function of miR-19b in human primary CD8^+^T cells has not been determined. In this study, the data revealed the pro-proliferative and anti-apoptotic role of miR-19b in CD8^+^T cells from both HCs and HIV-infected patients. These findings are consistent with data reported in mice (43, 44). In addition, we found that miR-19b significantly enhances the antiviral responses of CD8^+^T cells (i.e., secretion of IFN-γ and granzyme B after stimulation of the anti-CD3/CD28). It is evident that miR-19b plays an important role in the regulation of CD8^+^T cell responses against HIV. Through a bioinformatics analysis, we analyzed the cell signaling pathways involving miR-19b target genes and assessed the effect of PTEN downregulation on the function of miR-19b in CD8^+^T cells. Previous studies have shown that miR-19b downregulates the expression of the target gene PTEN. However, the involvement of PTEN in the regulation of CD8^+^T cell functions by miR-19b in HIV infection had not been investigated. This study confirmed that downregulation of PTEN antagonizes the effect of miR-19b inhibition on the function of CD8^+^T cells in HIV patients, suggesting that PTEN is one of the targets of miR-19b in CD8^+^T cells. Our results indicate that miR-19b influences the low viral loads in LTNP-Ls through the regulation of CD8^+^T cells in HIV infection. Furthermore, we found that miR-19b directly inhibits viral production in *in-vitro* HIV-infected T cell lines and primary CD4^+^T cells. Previous studies have shown that miR-19b inhibits the replication of hepatitis B virus (45, 46). However, its effect on the viral production of HIV has not been reported. Our study demonstrated that miR-19b inhibits the production of HIV in T cells. Previous study showed that overexpressing PTEN enhanced HIV-1 expression by inhibiting PI3K (47). We found that miR-19b was increased in LTNPs with sustained viral control. Because overexpression of miR-19b inhibits PTEN expression, we postulated that PTEN might be involved in the viral control by miR-19b in LNTP-Ls. Our study emphasized the need to further examine the mechanism of direct inhibition of HIV in detail. We found that the expression of miR-19b was decreased in sorted CD8^+^T cells and CD4^+^T cells in LTNP-Hs compared with that in LTNP-Ls. The current data highlighted the importance of miR-19b in HIV infection, promoting CD8^+^T cell function and inhibiting viral production. These results provide potential targets for improved immune intervention.

In summary, this study provided the first comprehensive overview of the expression of miRNA in LTNP-Hs and LTNP-Ls. The investigation identified miR-19b as a highly expressed miRNA in LTNP-Ls, contributing to low viral load in LTNPs through promotion of CD8^+^T cell function and inhibition of viral production. This study provides useful information for the exploration of new intervention paths in HIV infection.

## Author Contributions

HS, Z-NZ, and L-BY conceived and designed the experiments. L-BY performed the experiments. L-BY analyzed the data. C-BS, J-FZ, Y-JF, SQ, Y-JJ, J-JX, and H-BD contributed reagents, materials, and analysis tools. L-BY and Z-NZ wrote the article.

### Conflict of Interest Statement

The authors declare that the research was conducted in the absence of any commercial or financial relationships that could be construed as a potential conflict of interest.
