# Supplementary material for: Elevated Expression of miR-19b Enhances CD8+ T Cell Function by Targeting PTEN in HIV Infected Long Term Non-progressors With Sustained Viral Suppression
Source: Front Immunol. 2019 Jan 11;9:3140. doi: 10.3389/fimmu.2018.03140 (PMC6338066; doi:10.3389/fimmu.2018.03140)
Supplement: Supplementary file 1 [file Table_1.DOCX]

**Supplemental Material**

**Supplemental Table 1.** Demographic and clinical characteristics of LTNPs.

Characteristic LTNP-Hs LTNP-Ls

miRNA training cohort

n 6 3

Male, no. (%) 5 (83.33) 3 (100)

Age, years, mean (SD) 42.53 (6.34) 37.68 (3.68)

CD4, cells/µl, mean (SD) 712.67 (155.52) 911.67 (200.95)

CD8 cells/µl, mean (SD) 1144.50(202.18) 835.67(326.50)

VL, copies/ml, mean (SD) 16121.67 (20818.73) 124.00 (124.77)

Han ethnic, no. (%) 6(100) 3(100)

Transmission method former blood donation former blood donation

miRNA validation cohort

n 10 8

Male, no. (%) 8 (80) 3 (37.5)

Age, years, mean (SD) 51.63 (7.70) 49.06 (7.16)

CD4, cells/µl, mean (SD) 741.22 (257.64) 715.39 (179.33)

CD8, cells/µl, mean (SD) 1638.33(886.92) 1220.59(641.40)

VL, copies/ml, mean (SD) 22590.00 (29431.03) 228.25 (280.47)

Han ethnic, no. (%) 10(100) 8(100)

Transmission method former blood donation former blood donation
