# Supplementary material for: Elevated Expression of miR-19b Enhances CD8+ T Cell Function by Targeting PTEN in HIV Infected Long Term Non-progressors With Sustained Viral Suppression
Source: Front Immunol. 2019 Jan 11;9:3140. doi: 10.3389/fimmu.2018.03140 (PMC6338066; doi:10.3389/fimmu.2018.03140)
Supplement: Supplementary file 2 [file Table_2.DOCX]

**Supplemental Material**

**Supplemental Table 2** Demographic and clinical characteristics of TPs

| Characteristic | TPs |
| --- | --- |
| n | 7 |
| Male, no. (%) | 6 (100) |
| Age, years, mean (SD) | 30 (15) |
| Han ethnic, no. (%) | 6(100) |
| Antiretroviral therapy | none |
| CD4, cells/µl, mean (SD) | 676.47 (91.61) |
| CD8 cells/µl, mean (SD) | 1528.78 (712.59) |
| VL, copies/ml, mean (SD) | 4524.33 (6344.77) |
