# Supplementary material for: Elevated Expression of miR-19b Enhances CD8+ T Cell Function by Targeting PTEN in HIV Infected Long Term Non-progressors With Sustained Viral Suppression
Source: Front Immunol. 2019 Jan 11;9:3140. doi: 10.3389/fimmu.2018.03140 (PMC6338066; doi:10.3389/fimmu.2018.03140)
Supplement: Supplementary file 3 [file Table_3.DOCX]

**Supplemental Material**

**Supplemental Table 3.** Mimic, inhibitor and primer Sequences for experiments.

**Mimics** **Sense Sequence ( 5`- 3`) Antisense sense Sequence ( 5`- 3`)**

miR-19b UGUGCAAAUCCAUGCAAAACUGA AGUUUUGCAUGGAUUUGCACAU N.C UUCUCCGAACGUGUCACGUTT ACGUGACACGUUCGGAGAATT

**Inhibitor Sequence ( 5`- 3`)**

miR-19b UCAGUUUUGCAUGGAUUUGCACA

N.C CAGUACUUUUGUGUAGUACAA

**Gene Forward primer sequence ( 5`- 3`) Reverse primer sequence ( 5`- 3`)**

miR-19b TGTGCAAATCCATGCAAAACTGA mRQ 3’ Primer

miR-15a TAGCAGCACATAATGGTTTGTG mRQ 3’ Primer

miR-33 GTGCATTGTAGTTGCATTGCA mRQ 3’ Primer

U6 ATGGACTATCATATGCTTACCGTA mRQ 3’ Primer

PTEN ACCATAACCCACCACAGC CAGTTCGTCCCTTTCCAG

FBXO32 GAAGCGCTTCCTGGATGAGA GGAATCCAGAATGGCAGTTG

MAPK14 CGAAGATGAACTTTGCGAATG GCTTGGGCCGCTGTAATT

CCND2 TCCAAACTCAAAGAGACCAGC TTCCACTTCAACTTCCCCAG

GAPDH GCCTTCCGTGTCCCCACTGC CAATGCCAGCCCCAGCGTCA
