# Supplementary material for: Elevated Expression of miR-19b Enhances CD8+ T Cell Function by Targeting PTEN in HIV Infected Long Term Non-progressors With Sustained Viral Suppression
Source: Front Immunol. 2019 Jan 11;9:3140. doi: 10.3389/fimmu.2018.03140 (PMC6338066; doi:10.3389/fimmu.2018.03140)
Supplement: Supplementary file 4 [file Table_4.DOCX]

**Supplemental Material**

**Supplemental Table 4.** The list of regulated miRNAs in different groups.

|  |  |  |  |
| --- | --- | --- | --- |
| **microRNA ID** | **P.Val** | **adj P.Val** | **Log2FC** |
| Comparison of MicroRNA expression in LTNP-Ls and LTNP-Hs | | | |
| hsa-miR-674 | 5.00E-09 | 1.50E-06 | -6.322444 |
| hsa-miR-1277 | 1.32E-08 | 1.50E-06 | 7.183549 |
| hsa-miR-18b | 1.42E-08 | 1.50E-06 | -6.561737 |
| hsa-miR-721 | 1.73E-08 | 1.50E-06 | 7.060466 |
| hsa-miR-126-5p | 2.33E-08 | 1.62E-06 | -8.043949 |
| hsa-miR-95 | 3.45E-08 | 2.00E-06 | 7.591294 |
| hsa-miR-27b* | 5.69E-08 | 2.82E-06 | 6.202869 |
| hsa-miR-181d | 2.70E-07 | 1.17E-05 | 6.31269 |
| hsa-let-7g* | 7.08E-07 | 2.73E-05 | 7.283747 |
| hsa-miR-212 | 9.47E-07 | 3.29E-05 | -4.492149 |
| hsa-mir-453 | 1.06E-06 | 3.34E-05 | 5.506773 |
| hsa-miR-302b | 1.26E-06 | 3.65E-05 | 5.275118 |
| hsa-miR-16-2* | 2.79E-06 | 7.44E-05 | 4.653275 |
| hsa-miR-494 | 7.64E-06 | 1.89E-04 | 5.92591 |
| hsa-let-7g | 8.49E-06 | 1.95E-04 | -4.751614 |
| hsa-miR-200c | 8.98E-06 | 1.95E-04 | -9.18341 |
| hsa-miR-34b-3p | 1.10E-05 | 2.24E-04 | 3.957506 |
| hsa-miR-742 | 1.48E-05 | 2.86E-04 | 4.477559 |
| hsa-miR-7a* | 1.95E-05 | 3.55E-04 | -4.479374 |
| hsa-miR-29a | 2.42E-05 | 4.19E-04 | -4.433576 |
| hsa-miR-196a | 2.85E-05 | 4.71E-04 | 4.405273 |
| hsa-miR-98 | 3.73E-05 | 5.82E-04 | 4.622901 |
| hsa-miR-380-5p | 3.86E-05 | 5.82E-04 | 3.83904 |
| hsa-let-7f | 5.04E-05 | 7.28E-04 | -3.763006 |
| hsa-miR-520g | 7.24E-05 | 1.01E-03 | 3.820498 |
| hsa-miR-450-3p | 7.64E-05 | 1.01E-03 | 3.463509 |
| hsa-miR-690 | 7.83E-05 | 1.01E-03 | -4.541532 |
| hsa-miR-191* | 9.18E-05 | 1.14E-03 | 4.832613 |
| hsa-miR-340-5p | 0.000100 | 0.001201 | -2.941039 |
| hsa-miR-142-5p | 0.000135 | 0.001524 | 3.364152 |
| hsa-mir-214* | 0.000136 | 0.001524 | 5.885925 |
| hsa-miR-302c | 0.000152 | 0.001644 | 4.54752 |
| hsa-miR-508-3p | 0.000158 | 0.001659 | 3.404541 |
| hsa-miR-615-5p | 0.000244 | 0.002493 | 3.868699 |
| hsa-miR-30d | 0.000265 | 0.002631 | -3.280748 |
| hsa-miR-154 | 0.000304 | 0.002933 | 3.234764 |
| hsa-miR-34c | 0.000325 | 0.003046 | 3.099342 |
| hsa-miR-468 | 0.000360 | 0.00329 | 2.911727 |
| hsa-miR-526a | 0.000388 | 0.003449 | -3.097779 |
| hsa-miR-693-5p | 0.000399 | 0.00346 | -4.000644 |
| hsa-miR-677 | 0.000465 | 0.003938 | 3.442755 |
| hsa-miR-20a | 0.000566 | 0.004679 | 4.013713 |
| hsa-miR-302a | 0.000654 | 0.005276 | 4.340215 |
| hsa-miR-763 | 0.000715 | 0.005642 | 3.30676 |
| hsa-miR-26b* | 0.000761 | 0.005865 | 2.586165 |
| hsa-miR-181a | 0.001013 | 0.00764 | -2.622951 |
| hsa-miR-143 | 0.001081 | 0.00794 | 2.57524 |
| hsa-miR-17 | 0.001098 | 0.00794 | -2.857594 |
| hsa-miR-16-1* | 0.001172 | 0.008296 | 2.700822 |
| hsa-miR-195 | 0.001310 | 0.009089 | -2.571373 |
| hsa-miR-10a | 0.001399 | 0.00952 | -2.655695 |
| hsa-miR-208b | 0.001470 | 0.009812 | 3.445991 |
| hsa-miR-199b-5p | 0.001505 | 0.00985 | 2.385926 |
| hsa-miR-706 | 0.001536 | 0.009869 | -2.303394 |
| hsa-miR-182 | 0.001981 | 0.012501 | 2.336094 |
| hsa-miR-382 | 0.002083 | 0.012904 | -3.121864 |
| hsa-miR-141* | 0.003129 | 0.019048 | -2.203254 |
| hsa-miR-519a* | 0.003492 | 0.020892 | -1.939296 |
| hsa-miR-26a-2* | 0.003615 | 0.021262 | 2.404559 |
| hsa-miR-505 | 0.003882 | 0.022454 | 1.982804 |
| hsa-let-7a | 0.004273 | 0.02431 | -1.992083 |
| hsa-miR-450a-5p | 0.004948 | 0.027691 | 2.226329 |
| hsa-let-7d | 0.005074 | 0.027947 | 2.073109 |
| hsa-miR-33 | 0.005395 | 0.028852 | 2.087788 |
| hsa-miR-204 | 0.005405 | 0.028852 | 2.646827 |
| hsa-miR-15a | 0.005745 | 0.029985 | 1.933902 |
| hsa-miR-302d | 0.005790 | 0.029985 | 3.306004 |
| hsa-miR-19a | 0.005981 | 0.030523 | 1.979634 |
| hsa-miR-200c* | 0.006513 | 0.032756 | 2.205307 |
| hsa-miR-96 | 0.006714 | 0.032782 | 1.936107 |
| hsa-mir-106b* | 0.006725 | 0.032782 | 2.390397 |
| hsa-miR-31* | 0.006832 | 0.032782 | 1.802997 |
| hsa-miR-712* | 0.006896 | 0.032782 | 2.647929 |
| hsa-miR-411 | 0.007180 | 0.033669 | 2.74615 |
| hsa-miR-496 | 0.007942 | 0.036735 | 1.864958 |
| hsa-miR-139-3p | 0.008046 | 0.036735 | 6.699371 |
| hsa-miR-19b | 0.009951 | 0.044843 | 1.827089 |
| hsa-miR-449a | 0.010767 | 0.047898 | 1.93159 |
| Comparison of MicroRNA expression in LTNP-Hs and HCs | | | |
| hsa-miR-18b | 3.91E-09 | 9.55E-07 | -6.738833 |
| hsa-miR-674 | 6.03E-09 | 9.55E-07 | -6.864827 |
| hsa-miR-1277 | 8.26E-09 | 9.55E-07 | 8.000489 |
| hsa-miR-721 | 1.51E-08 | 1.31E-06 | 7.153305 |
| hsa-miR-95 | 7.57E-08 | 4.62E-06 | 7.369077 |
| hsa-miR-181d | 7.99E-08 | 4.62E-06 | 7.820917 |
| hsa-miR-302b | 1.19E-07 | 5.65E-06 | 5.728282 |
| hsa-miR-126-5p | 1.30E-07 | 5.65E-06 | -7.354476 |
| hsa-miR-200c | 1.52E-06 | 5.84E-05 | -9.691913 |
| hsa-miR-450-3p | 2.60E-06 | 9.01E-05 | 4.429719 |
| hsa-miR-191* | 3.86E-06 | 1.22E-04 | 6.349298 |
| hsa-miR-7a* | 4.86E-06 | 1.41E-04 | -4.706985 |
| hsa-miR-98 | 5.93E-06 | 1.58E-04 | 5.725913 |
| hsa-miR-494 | 7.32E-06 | 1.71E-04 | 6.207323 |
| hsa-miR-196a | 7.39E-06 | 1.71E-04 | 5.218628 |
| hsa-mir-453 | 8.08E-06 | 1.75E-04 | 4.97422 |
| hsa-miR-212 | 1.90E-05 | 3.89E-04 | -3.374489 |
| hsa-miR-615-5p | 2.33E-05 | 4.49E-04 | 5.791258 |
| hsa-let-7g | 3.39E-05 | 5.89E-04 | -4.347414 |
| hsa-miR-677 | 4.99E-05 | 8.24E-04 | 3.827465 |
| hsa-miR-526a | 9.78E-05 | 1.54E-03 | -3.388784 |
| hsa-miR-520g | 2.16E-04 | 3.12E-03 | 3.952416 |
| hsa-miR-20a | 2.33E-04 | 3.24E-03 | 4.374478 |
| hsa-miR-693-5p | 3.05E-04 | 4.07E-03 | -3.739972 |
| hsa-miR-468 | 3.22E-04 | 4.14E-03 | 3.25221 |
| hsa-let-7g* | 3.59E-04 | 4.45E-03 | 5.704994 |
| hsa-miR-763 | 6.73E-04 | 8.06E-03 | 3.215593 |
| hsa-miR-142-5p | 7.72E-04 | 8.93E-03 | 2.4907 |
| hsa-miR-208b | 0.001169 | 0.012674 | 3.425796 |
| hsa-miR-17 | 0.001650 | 0.016363 | -2.918148 |
| hsa-miR-690 | 0.002166 | 0.019783 | -3.729444 |
| hsa-miR-27b* | 0.002227 | 0.019818 | 3.973438 |
| hsa-miR-449a | 0.002445 | 0.021214 | 2.570116 |
| hsa-miR-31* | 0.002922 | 0.024143 | 2.146613 |
| hsa-miR-340-5p | 0.003066 | 0.024393 | -2.290302 |
| hsa-miR-182 | 0.003093 | 0.024393 | 2.272174 |
| hsa-miR-200c* | 0.004237 | 0.031281 | 2.406238 |
| hsa-miR-30d | 0.004893 | 0.033958 | -2.146467 |
| hsa-miR-382 | 0.005089 | 0.033958 | -2.542852 |
| hsa-miR-181a | 0.006215 | 0.039208 | -2.06423 |
| hsa-miR-34c | 0.006461 | 0.039252 | 2.448121 |
| hsa-miR-505 | 0.006561 | 0.039252 | 1.77041 |
| hsa-miR-302c | 0.007392 | 0.043281 | 5.691587 |
| hsa-miR-706 | 0.007484 | 0.043281 | -2.035087 |
| hsa-miR-195 | 0.008976 | 0.049437 | -1.887895 |
| hsa-miR-712* | 0.009178 | 0.049763 | 3.217162 |
| hsa-let-7f | 0.009344 | 0.049884 | -2.388044 |
| hsa-miR-411 | 0.009909 | 0.052100 | 2.415366 |
| hsa-miR-26a-2* | 0.010388 | 0.053184 | 2.039987 |
| hsa-miR-29a | 0.010422 | 0.053184 | -2.777026 |
| hsa-miR-519a* | 0.010886 | 0.053641 | -1.661392 |
| hsa-miR-10a | 0.011060 | 0.053641 | -2.044559 |
| hsa-miR-496 | 0.016145 | 0.065816 | 1.681346 |
| hsa-miR-141* | 0.016194 | 0.065816 | -1.815262 |
| hsa-miR-199b-5p | 0.016312 | 0.065816 | 1.70063 |
| hsa-miR-34b-3p | 0.019100 | 0.071732 | 2.307227 |
| hsa-miR-19a | 0.027903 | 0.097802 | 1.829812 |
| hsa-let-7a | 0.029270 | 0.100561 | -1.762936 |
| hsa-miR-450a-5p | 0.029862 | 0.101588 | 1.6079 |
| hsa-miR-96 | 0.037649 | 0.119854 | 2.198625 |
| hsa-miR-204 | 0.047956 | 0.141878 | 1.627917 |
| Comparison of MicroRNA expression in LTNP-Ls and HCs | | | |
| hsa-miR-139-3p | 0.014118 | 0.587462 | 19.400135 |
| hsa-mir-214* | 0.007207 | 0.500149 | 3.38961 |
| hsa-miR-380-5p | 0.000923 | 0.160156 | 2.924042 |
| hsa-miR-302a | 0.002888 | 0.334010 | 2.589815 |
| hsa-miR-508-3p | 0.000665 | 0.160156 | 2.573952 |
| hsa-miR-16-1* | 0.00614 | 0.500149 | 2.291631 |
| hsa-miR-742 | 0.025995 | 0.751691 | 1.841552 |
| hsa-miR-143 | 0.012363 | 0.587462 | 1.692223 |
| hsa-miR-154 | 0.032449 | 0.823918 | 1.40782 |
| Comparison of MicroRNA expression in LTNP-Ls and TPs | | | |
| hsa-miR-16-2* | 0.000122 | 0.007050 | 4.224223 |
| hsa-mir-106b* | 0.002939 | 0.033998 | 2.472024 |
| hsa-miR-302d | 0.024247 | 0.133549 | 2.143917 |
| hsa-miR-26b* | 0.035648 | 0.169451 | 1.687328 |
| hsa-let-7d | 0.040363 | 0.182019 | 1.617907 |
|  |  |  |  |
