# Supplementary material for: Elevated Expression of miR-19b Enhances CD8+ T Cell Function by Targeting PTEN in HIV Infected Long Term Non-progressors With Sustained Viral Suppression
Source: Front Immunol. 2019 Jan 11;9:3140. doi: 10.3389/fimmu.2018.03140 (PMC6338066; doi:10.3389/fimmu.2018.03140)
Supplement: Supplementary file 5 [file Table_5.DOCX]

**Supplemental Material**

**Supplemental Table 5.** Demographic and clinical characteristics of HIV-1 infected donors

| Characteristic | HIV-1 infected donors |
| --- | --- |
| n | 7 |
| Male, no. (%) | 6 (85.72) |
| Age, years, mean (SD) | 30 (5) |
| Han ethnic, no. (%) | 7(100) |
| Antiretroviral therapy | none |
| CD4, cells/µl, mean (SD) | 220.29 (173.32) |
| CD8 cells/µl, mean (SD) | 706.57(320.54) |
| VL, copies/ml, mean (SD) | 129642.86 (255910.05) |
| Co- infection |  |
| HBV | none |
| HCV | none |
| Co-morbidities | none |
| Time since diagnosis, days, mean (Range) | 286(5-1728) |
