# Supplementary figures and images for: Elevated Expression of miR-19b Enhances CD8+ T Cell Function by Targeting PTEN in HIV Infected Long Term Non-progressors With Sustained Viral Suppression
Source: Front Immunol. 2019 Jan 11;9:3140. doi: 10.3389/fimmu.2018.03140 (PMC6338066; doi:10.3389/fimmu.2018.03140)

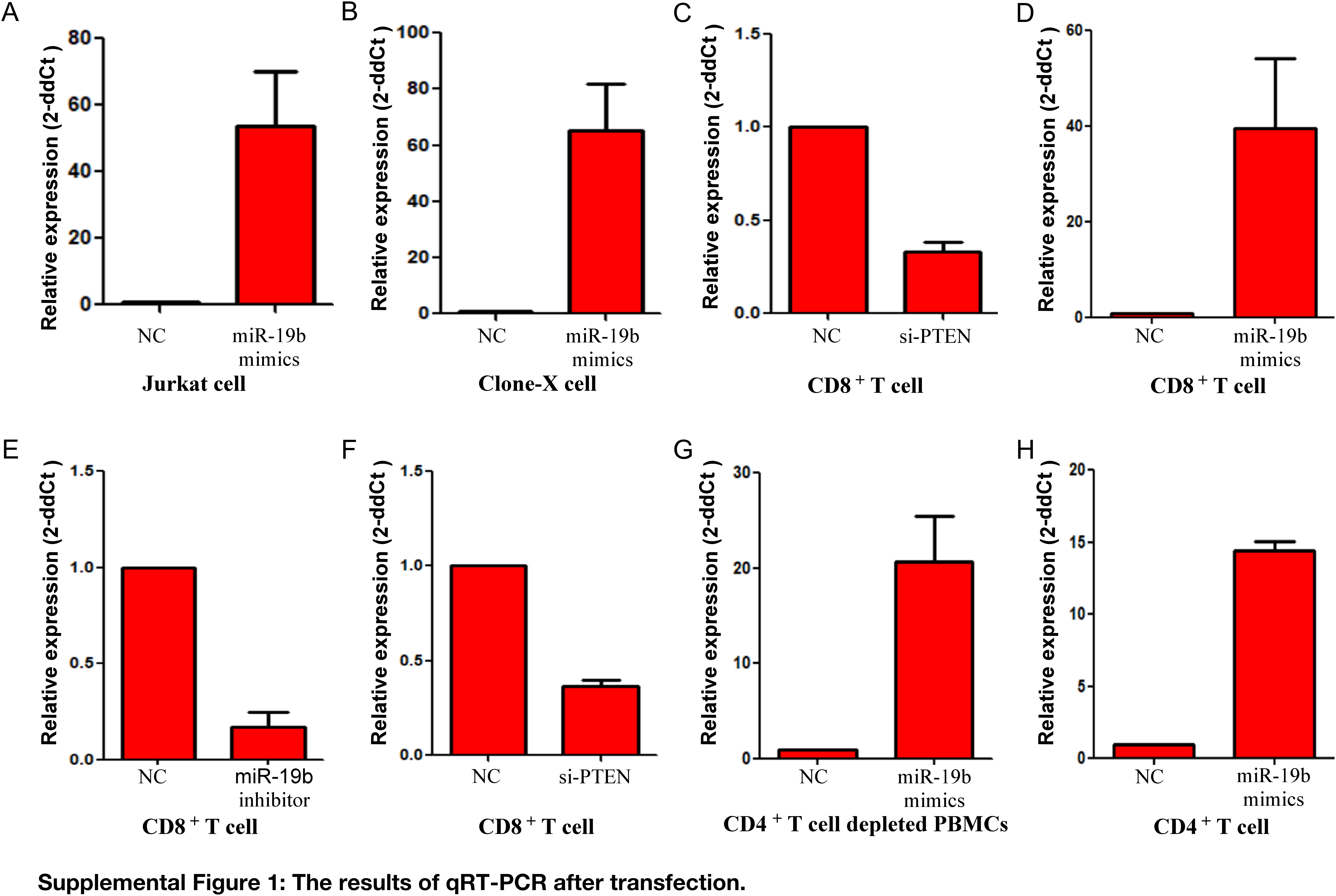

Supplement: Supplementary file 6 [file Image_1.tif]
